# Mechanical ventilation with heliox in an animal model of acute respiratory distress syndrome

**DOI:** 10.1186/2197-425X-2-8

**Published:** 2014-02-06

**Authors:** Charlotte J Beurskens, Hamid Aslami, Friso M de Beer, Joris JTH Roelofs, Margreeth B Vroom, Nicole P Juffermans

**Affiliations:** Laboratory of Experimental Intensive Care and Anaesthesiology, Academic Medical Center, University of Amsterdam, Meibergdreef 9, Amsterdam, 1105 AZ the Netherlands; Department of Intensive Care, Academic Medical Center, University of Amsterdam, Meibergdreef 9, Amsterdam, 1105 AZ the Netherlands; Department of Pathology, Academic Medical Center, University of Amsterdam, Amsterdam, 1012 WX the Netherlands

**Keywords:** Intensive care medicine, Heliox, Mechanical ventilation, ARDS, Animal model

## Abstract

**Background:**

Heliox has a lower density and higher diffusion capacity compared to oxygen-in-air. We hypothesized that heliox ventilation allows for a reduction in minute volume ventilation and inspiratory pressures needed for adequate gas exchange in an animal model of an acute lung injury.

**Methods:**

After intratracheal instillation of lipopolysaccharide (10 mg/kg), adult rats were randomized to ventilation with either a gas mixture of helium/oxygen (50:50%) or oxygen/air (50:50%). They were mechanically ventilated according to the ARDSnet recommendations with tidal volumes of 6 ml/kg and monitored with a pneumotachometer. Bronchoalveolar lavage fluid was analyzed for markers of lung injury, and embedded lung sections were histologically scored for lung injury.

**Results:**

Heliox limited the increase in driving pressures needed to achieve preset tidal volumes, with a concomitant decrease in loss of compliance. Heliox did neither allow for reduced minute volume ventilation in this model nor improve gas exchange. Also, heliox did not reduce lung injury.

**Conclusions:**

Heliox modestly improved respiratory mechanics but did not improve lung injury in this rat model of acute respiratory distress syndrome.

## Background

Obstructed airways with increased airway resistance and high inspiratory pressures needed for adequate gas exchange are common features in acute respiratory distress syndrome (ARDS) [1–3]. A limited tidal volume ventilation of 6 ml/kg is proven to be beneficial in ARDS [4]. However, application of limited tidal volume ventilation can often not be achieved in ARDS because of hypoxemia and acidosis [5, 6]. Also, application of relatively low plateau pressures and driving pressures can already be harmful [7, 8]. Thereby, mechanical ventilation may aggravate ARDS, the extent of which is affected by the intensity of ventilation. In ARDS, higher minute volumes are needed to compensate for increased oxygen demand and carbon dioxide production, increasing the risk of additional injury by mechanical ventilation. Therefore, in ARDS, adjunctive therapies which allow for less invasive ventilation are worth exploring.

Helium is an inert noble gas with a lower density compared to nitrogen. Thereby, mechanical ventilation using a gas mixture of oxygen and helium (heliox) can reduce turbulent gas flow and establish a more laminar flow [9]. This biochemical feature enables a reduction in the work of breathing in patients with obstructed airflow due to increased airway resistance during exacerbations of asthma and COPD [10]. Another important feature of heliox is the increased diffusion of CO_2_ compared to air [9]. Consequently, heliox decreases inspiratory pressures required to establish a set gas flow and to enable gas exchange in distant alveoli [11, 12].

The use of heliox has been evaluated in pediatric animal models of ARDS induced by oleic acid or saline [13–15], showing that heliox ventilation improved gas exchange during high-frequency oscillatory ventilation. We recently showed that heliox improves CO_2_ removal and decreases driving pressures in patients who are mechanically ventilated according to the recommended protective strategy, as well as in an animal model of lung injury inflicted by high tidal volume ventilation [16, 17].

In this study, we investigated the effects of heliox in an adult lung injury model. We hypothesized that the use of heliox reduces minute volume ventilation and inspiratory pressures with improved compliance. Furthermore, we hypothesized that the use of heliox facilitates CO_2_ elimination during protective mechanical ventilation. Although without a clear mechanism known, heliox showed anti-inflammatory effects in ARDS models in previous studies [14, 18], so the effect on the inflammatory response was also investigated.

## Methods

### Animal study design

The animal care and use committee of the Academic Medical Center, University of Amsterdam, Netherlands approved this study. Animal procedures were carried out in compliance with Institutional Standards for Use of Animal Laboratory Animals.

### Induction of lung injury, anesthesia, instrumentation, and mechanical ventilation

Male Sprague–Dawley rats (Harlan, The Hague, The Netherlands), weighing 350 to 400 g, were randomized into four experimental groups. Two groups were anesthetized using a transoral miniature nebulizer under light anesthesia (97% oxygen with 3% isoflurane) and intratracheally instilled with 1 mg/kg of *Escherichia coli* lipopolysaccharide (LPS) (L4131, 7.5 mg/kg, Sigma Aldrich, Steinheim, Germany). Control groups received no instillation. Two hours after LPS instillation, the animals were anesthetized by intraperitoneal injection of 90 mg/kg ketamine (Nimatek®; Eurovet Animal Health BV, Bladel, the Netherlands), 0.125 mg/kg dexmedetomidine (Dexdomitor®; Orion Pharma, Espoo, Finland), and 0.05 mg/kg atropine (atropine sulfate; Centrafarm BV, Etten-Leur, the Netherlands). Via a tail vein Venflon cannula, anesthesia was maintained by infusion of 10 mg/ml ketamine at 2.7 ml/h. A solution of saline and 4.2 mg/ml bicarbonate (Fresenius Kabi Nederland BV, Hertogenbosch, the Netherlands) was administered at 2.5 ml/h.

A tracheotomy was performed and a metal cannula was inserted into the trachea. Two sutures were placed around the exposed part of the trachea into which the cannula was tied down thoroughly. The cannula was then connected to a ventilator (Servo 900C, Siemens, Upplands Vasby, Sweden). The ventilators were calibrated for the heliox gas mixture according to the instruction of the manufacturer using a pressure reduction valve to allow the high-pressure of the heliox tank to be reduced to safe and usable pressures for ventilation (Linde Gas Therapeutics, Eindhoven, the Netherlands).

Hemodynamic parameters were monitored by inserting a polyethylene heparinized-saline (1:1,000)-filled catheter into the right carotid artery (Braun, Melsungen, Germany) that was connected to a monitor (Siemens SC900, Danvers, MA, USA). Temperature was monitored rectally (Ama-digit ad 15th, Amarell, Kreuzwertheim, Germany) and maintained at 37°C by a thermo mattress.

The rats were ventilated in a pressure-controlled mode for 4 h, with either heliox (technical gas 50% oxygen; 50% helium; blended by Linde Gas Therapeutics) or 50% oxygen-in-air gas mixture. In total, 32 animals were studied of which 16 received heliox (8 LPS, 8 healthy controls) and 16 received 50% oxygen-in-air gas mixture (8 LPS, 8 healthy controls).

Lung protective (LP) ventilation was maintained, according to a fixed protocol, by applying 6 ml/kg and 5 cm H_2_O positive end-expiratory pressure (PEEP). FiO_2_ was set at 50% with an inspiration to expiration ratio of 1:2 and adjustment of respiratory rate to maintain arterial PaCO_2_ within 4.5 to 6.0 kPa, according to hourly drawn arterial blood gases (RAPIDlab 865 blood gas analyzer, Bayern, Mijdrecht, the Netherlands).

Tidal volumes were strictly maintained using a pneumotachometer (Hugo Sachs Elektronik, Harvard apparatus, March-Hugstetten, Germany) specific for rats. The pneumotachometer is a transducer for airflow measurement, placed between the metal cannula and the ventilator. For both heliox and oxygen ventilation, the pneumotachometer was calibrated using a 1-ml syringe according to the manufacturer's instruction. Tidal volumes were recorded using respiration software (HSE-BDAS basic data acquisition, Harvard apparatus, March-Hugstetten, Germany) and displayed on a computer screen throughout the whole experiment. We set a pressure controlled ventilation mode and started with an inspiratory pressure of 15 cm H_2_O. The tidal volume was targeted by adjusting the inspiratory pressure [17, 19–21].

The inspiratory pressures were recorded every hour. The driving pressure was calculated by inspiratory pressure minus PEEP. Compliance was calculated by dividing the tidal volume per kilogram by the driving pressure. Minute volume was calculated in milliliter per minute by multiplying respiratory rate with the measured tidal volume.

### Inflammation measurements

After 4 h of mechanical ventilation, the rats were bled and plasma was centrifuged at 1,800 *× g* for 10 min at 4°C. The lungs were removed *en bloc* and the right lung was ligated. A bronchoalveolar lavage was done by flushing the left lung three times with 2.0 ml NaCl, yielding approximately 5.5 to 6.0 ml of bronchoalveolar lavage fluid (BALF).

In BALF, the cells were counted using a hematocytometer (Z2 Coulter Particle Counter, Beckman Coulter Corporation; Hialeah, Florida, USA). After centrifugation of BALF (300 *× g* for 10 min at 4°C), protein levels were measured (Oz Biosciences, Marseille, France) and levels of interleukin (IL)-1β, IL-6, cytokine-induced neutrophil chemoattractant 3 (CINC-3), and tumor necrosis factor alpha (TNF-α) were determined by ELISA in BALF and blood, according to instructions of the manufacturer (R&D Systems, Abingdon, UK).

The upper lobe of the right lung was fixed in 1% buffered formaldehyde and subsequently embedded in paraffin and afterwards cut into 5-μm-thick sections. The lung sections were fixed on glass slides and stained with hematoxylin and eosin and were analyzed by a pathologist, who was blinded to group identity, with the use of total histology score. This score consists of several parameters, including interstitial inflammation, endothelialitis, edema, bronchitis, thrombus, and pleuritis. All parameters were scored on a scale of 0 to 4: 0 for normal lungs, 1 for <25% lung involvement, 2 for 25% to 50% involvement, 3 for 50% to 75% involvement, and 4 for >75% lung involvement. The total histology score was calculated as the sum score of these parameters, with a maximum of 24. Furthermore, the pathologist was asked to choose one representative illustration per group.

### Statistical analysis

To compare time points (*T* = 0 vs. *T* = 4) within the same subject, a paired *t* test with Bonferroni correction was used if data were normally distributed, or Wilcoxon signed rank test in case of non-normal distribution. The effect of heliox versus oxygen-in-air at specific time points was compared using a one-way ANOVA or Kruskal-Wallis test, with either a Bonferroni's or Dunn's multiple comparison test, depending on the distribution of the data. Statistical significance was considered to be at *P* < 0.05 or at *P* < 0.0125 after Bonferroni correction. Data are expressed as mean ± SD.

## Results

All animals survived our experimental protocol. In animals receiving LPS, the mean arterial pressure dropped over time (162 ± 19 to 128 ± 18 mmHg), whereas it remained constant in the healthy controls. Heliox did not affect blood pressure or heart rate compared to the oxygen-in-air-ventilated animals.

### LPS induced acute lung injury

**Figure 1:**
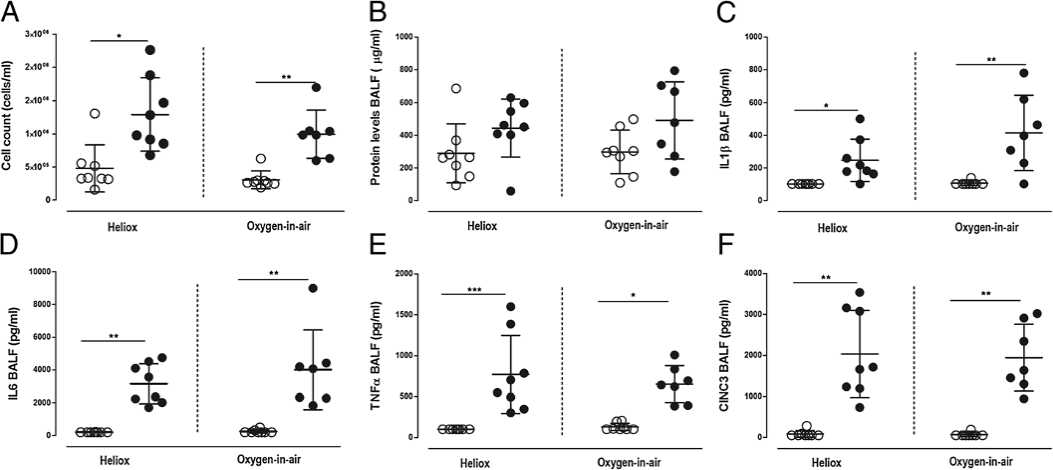
**Inflammatory parameters in an animal model of** **LPS-induced lung injury and healthy controls.** The animals were ventilated with heliox or oxygen-in-air (*N* = 8 per group). **(A)** Cell count. **(B)** Protein levels. **(C)** IL-1β levels. **(D)** IL-6 levels. **(E)** TNF-α levels. (**F**) CINC-3 levels in BALF. Healthy animals are marked by open dots and LPS animals by black dots. Data are presented as mean ± SD. *P < 0.05; **P < 0.01; ***P < 0.001.

**Figure 2:**
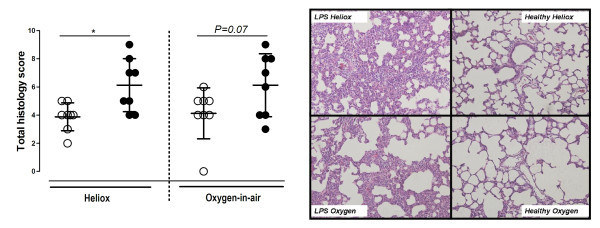
**Total histology score in an animal model of LPS-induced lung injury and healthy controls.** The model was ventilated with heliox or oxygen-in-air (*N* = 8 per group). Healthy animals are marked by open dots and LPS animals by black dots. Data are mean ± SD. *P < 0.05; **P < 0.01.

The instillation of LPS intratracheally resulted in a sufficient model of acute lung injury, based on the guidelines described by the American Thoracic Society [22]. The inflammatory response, one of the main features that characterize acute lung injury in animal models, was evidenced by increased pulmonary cell influx in the BALF (Figure 1A) and an increase in BALF cytokine levels of IL-1β, IL-6, TNF-α, and CINC-3 (Figure 1C,D,E,F). The protein levels in BALF were not significantly different due to a large variation (Figure 1B). Another important marker of lung injury in animals is the histological evidence of tissue injury, which in our study is measured by the total histopathology and which also increased due to LPS instillation (Figure 2). The pictures, chosen by the pathologist, show a representative example of each intervention group, showing interstitial inflammation, endothelialitis, edema, bronchitis, and pleuritis produced by the intratracheal LPS instillation.

**Figure 3:**
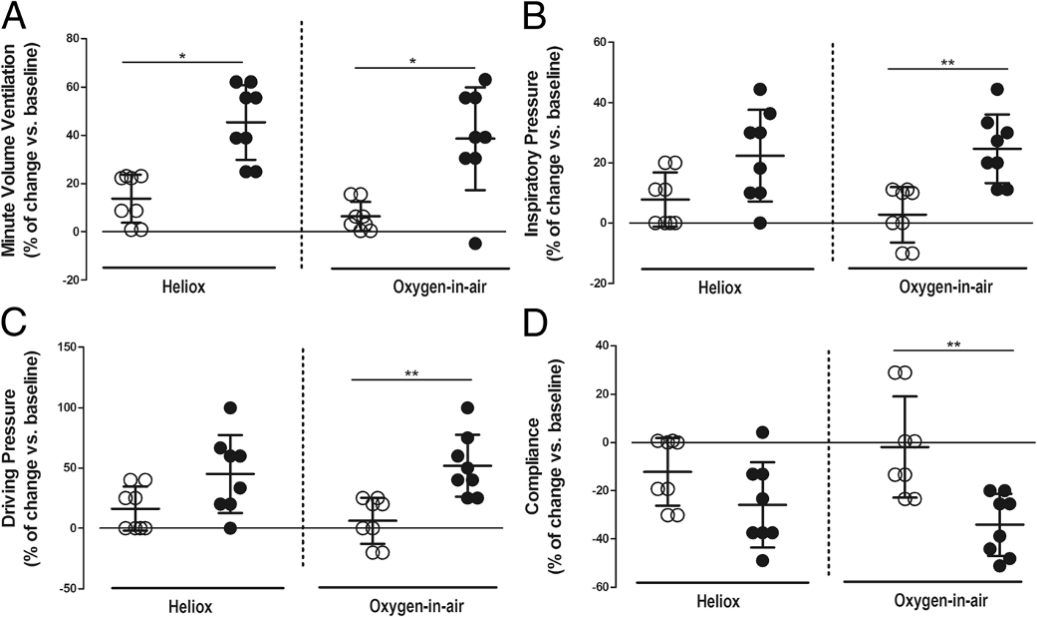
**Ventilatory parameters in an animal model of LPS–induced lung injury; changes versus baseline. (A)** Minute volume ventilation (ml/min), **(B)** inspiratory pressures (cm H_2_O), **(C)** driving pressures (cm H_2_O), and **(D)** compliance (μl/cm H_2_O) in an animal model of LPS-induced lung injury and healthy controls, ventilated with heliox or oxygen-in-air (*N* = 8 per group). Healthy animals are marked by open dots and LPS animals by black dots. Data are presented as mean ± SD. *P < 0.05; **P < 0.01.

The gas exchange at baseline and after 4 h of lung protective mechanical ventilation with heliox or oxygen-in-air was also affected by LPS instillation. Baseline pH showed a significant decrease with a concomitant increase in PaCO_2_ levels in LPS-challenged animals compared to healthy controls (Table 1). These differences in gas exchange disappeared during the experiment due to adjustments in respiratory rates. The applied respiratory rate needed to keep PaCO_2_ within predefined limits (4.5 to 6.0 kPa) increased during mechanical ventilation in both LPS-treated groups, yielding an increased percentage of change in minute volume ventilation versus the baseline measurement (Figure 3A). Inspiratory pressures applied to target tidal volumes of 6 ml/kg were also adjusted during the experiment, resulting in higher inspiratory and driving pressures in LPS-treated animals compared to healthy controls, with a significant increase in the animals ventilated with oxygen only (Figure 3B,C). A concomitant decrease in percentage change of compliance was seen in LPS-treated animals compared to healthy controls, with again a significant increase in the animals ventilated with oxygen (Figure 3D).

**Table 1:** **Gas exchange in an animal model of LPS–induced lung; ventilated with heliox or oxygen-in-air**

	Time (h)	Healthy		LPS	
		Oxygen-in-air	Heliox	Oxygen-in-air	Heliox
pH	0	7.44 ± 0.04	7.42 ± 0.03	7.35 ± 0.05^a^	7.34 ± 0.08^b^
	4	7.33 ± 0.04^c^	7.36 ± 0.03^d^	7.30 ± 0.06	7.30 ± 0.08
pCO_2_ (kPa)	0	4.38 ± 0.7	4.86 ± 0.6	5.78 ± 0.6^a^	6.10 ± 1.4^b^
	4	4.99 ± 0.4	5.81 ± 0.9	5.84 ± 0.77	5.75 ± 1.29
pO_2_ (kPa)	0	34.2 ± 1.8	31.7 ± 1.5	33.2 ± 1.7	32.3 ± 3.3
	4	31.7 ± 2.5	30.8 ± 2.8	28.5 ± 3.9	28.8 ± 3.4

^a^Oxygen-in-air LPS *T* = 0 vs. oxygen-in-air healthy *T* = 0, significant after Bonferroni's multiple comparison test (*P* < 0.01); ^b^Heliox LPS *T* = 0 vs. heliox healthy *T* = 0, significant after Bonferroni's multiple comparison test (*P* < 0.01); ^c^Oxygen-in-air healthy *T* = 0 vs. oxygen-in-air healthy *T* = 4, significant after Bonferroni correction (*P* < 0.0125); ^d^Heliox healthy *T* = 0 vs. heliox healthy *T* = 4, significant after Bonferroni correction (*P* < 0.0125). Data are presented as mean ± SD. *N* = 8 per group.

### Effects of heliox

Heliox did not result in a reduction of inflammatory parameters when compared to the oxygen-in-air ventilation. No effect was seen in the pulmonary neutrophil influx, protein, or cytokine levels in BALF (Figure 1A,B,C,D,E,F) or in the total histology score in the lung (Figure 2). Also, use of heliox did not alter gas exchange (Table 1). Comparing the change versus baseline, heliox did not influence minute volume ventilation needed for adequate gas exchange compared to oxygen-in-air ventilation (Figure 3A). Heliox did however abrogate the increase in inspiratory and driving pressures compared to baseline needed to generate tidal volumes of 6 ml/kg in LPS-treated animals compared to oxygen-in-air (Figure 3B,C). Concomitantly, the relative decrease in compliance in LPS-treated animals was abrogated with heliox ventilation versus oxygen-in-air ventilation (Figure 3D).

## Discussion

In this animal model of induced acute lung injury due to LPS instillation, heliox abrogated the increase in inspiratory and driving pressures needed to target preset tidal volumes over time. Furthermore, heliox diminished the decrease in compliance compared to baseline in LPS-treated animals. In contrast to our hypothesis, heliox did not allow for a reduction in minute volume ventilation during lung-protective mechanical ventilation. Moreover, heliox ventilation showed no effect on neither gas exchange or on lung inflammation. Thereby, our results are somewhat in contrast to studies investigating heliox ventilation in pediatric models as well as to findings in our own animal model of ventilator-induced lung injury, where heliox improved CO_2_ removal [13–17].

There may be several explanations for the absence of a beneficial effect of heliox in our model. Within our study design, we strictly regulated tidal volumes at 6 ml/kg. This study design was chosen to reduce confounders, influencing our results. With this design, we believe that any measured effect of heliox on lung injury could be ascribed to heliox only and not to a difference in tidal volumes. This distinction is important, because as a result of a more laminar flow pattern compared to oxygen-in-air ventilation, the mechanism by which heliox improves CO_2_ removal might be by an increased tidal volume delivery. In line with this, it was shown that during high-frequency oscillatory ventilation in an animal ARDS model, heliox did not alter gas exchange if tidal volume was kept constant [23]. If the findings would also be applicable during protective mechanical ventilation, based on the characteristics of heliox, we hypothesized a decrease in inspiratory pressures needed to generate a set tidal volume, as found before [12]. However, our effects on inspiratory and driving pressures, as well as the compliance, were only modest. Since we cannot exclude that heliox may be beneficial by allowing a further reduction of tidal volumes, which was shown before to increase protection in ARDS [24–26], the choice for strictly keeping tidal volumes at 6 ml/kg in these experiments limits the interpretation of our results.

An alternative explanation for the differences with previous studies may be related to differences in ventilation modes. The previous studies that showed a beneficial effect of heliox were all performed in pediatric animal models, mostly during high-frequency oscillatory ventilation [13–15]. This same mode was also used in infants, showing a decreased airway resistance during heliox ventilation [27].

Differences may also relate to the underlying disease state. Whereas airway obstruction is present in our model of acute lung injury, the severity of airway obstruction is far less compared to asthma, respiratory syncytial virus, or other disease states in which hyper-reactivity of bronchi plays an important role [27–29]. In line with this, in a test lung, the effect of heliox on reducing the inspiratory effort was shown to be dependent on the kind of obstruction and severity [30]. Our model may also have been a too mild model of lung injury, in which beneficial effects may be hard to tease out. However, our model is clinically relevant and represents the most important parameters of acute lung injury in animals [22].

The anti-inflammatory effects of heliox, previously reported in ARDS models [14, 18], could not be reproduced in our model. A clear mechanism on how heliox can affect lung injury markers and histology scoring is not known; however, in healthy volunteers helium resulted in an attenuated expression of inflammatory cell surface markers on leukocytes and platelets in blood [31]. It is postulated that the influence of helium might be via the cell-mediated immunity [32]. Furthermore, ventilation with heliox might lower shear stress and barotraumas and therefore have an anti-inflammatory effect [14]. However, our results indicate that there is no direct anti-inflammatory effect due to heliox ventilation.

## Conclusions

Heliox modestly limited the increase in inspiratory and driving pressures applied to target preset tidal volumes, with a concomitant restraint of the reduced compliance between LPS-treated animals and healthy controls. However, heliox ventilation did neither allow for lower minute volume ventilation nor did it have an effect on gas exchange, lung inflammation, or lung damage in an animal model of acute lung injury.
